# Management of Patients Undergoing Chemotherapy in Conservative Dentistry

**DOI:** 10.7759/cureus.117171

**Published:** 2026-09-29

**Authors:** Boutaina Derras, Nissrin Bassim

**Affiliations:** 1 Conservative Dentistry and Endodontics, Faculty of Dental Medicine, Mohammed V University in Rabat, Rabat, MAR; 2 Odontology, Military Hospital Mohammed V, Rabat, MAR

**Keywords:** cancer, chemotherapy, conservative dentistry, dental management, endodontics

## Abstract

The rising number of new cancer cases means that dentists are increasingly treating patients undergoing cancer treatments such as chemotherapy. Certain chemotherapy drugs can cause side effects in the oral cavity, which may significantly impair patients’ quality of life. Indeed, chemotherapy can have oral and dental repercussions with varied clinical manifestations. Their frequency and severity may depend on the treatment protocols used, the duration of treatment, any combination with radiation therapy, and the condition of the oral cavity before the start of treatment.

The aim of this narrative review is to summarize the current evidence and international recommendations regarding the prevention and management of oral complications associated with chemotherapy, with a particular focus on conservative dentistry and endodontic treatment before, during, and after chemotherapy.

Dentists therefore play a vital role in the multidisciplinary management of these patients. A thorough understanding of the risks associated with chemotherapy enables the implementation of preventive measures and appropriate conservative and endodontic treatment before, during, and after anticancer therapy, thereby reducing oral complications and improving patients’ quality of life.

## Introduction and background

Cancer is one of the leading causes of morbidity and mortality worldwide. As the second leading cause of death globally, cancer accounts for approximately 10 million deaths per year. Nearly one in six deaths worldwide is due to cancer, with approximately 19.3 million new cases in 2020 [1].

Chemotherapy forms part of the therapeutic arsenal for the management of patients with cancer. It uses anticancer (cytotoxic) drugs to destroy cancer cells. These drugs also affect healthy cells, causing general and oral side effects [2]. These may include mucositis, xerostomia, dysgeusia, increased susceptibility to oral infections, bleeding, and dental caries. Most side effects subside once treatment has finished. Chemotherapy may be administered as the primary treatment or following other treatments to reduce the risk of cancer recurrence. It may also be used to shrink a tumor before surgery or radiotherapy [2].

Apart from minor oral side effects, the risks of infection and bleeding remain the most serious concerns during and after chemotherapy. It is therefore important that the oncologist and dentist are aware of the risks involved and inform patients of these risks to ensure better prevention and management before, during, and after chemotherapy.

The aim of this narrative review was to summarize the current evidence and international guidelines regarding the prevention and management of oral complications associated with chemotherapy and to provide practical recommendations for conservative and endodontic treatment before, during, and after chemotherapy.

Literature search

This article is a narrative review. A literature search was conducted using PubMed/MEDLINE and Google Scholar. The literature considered included peer-reviewed original studies, narrative and literature reviews, clinical guidelines, professional recommendations, and clinical practice statements addressing chemotherapy-related oral complications and dental management.

Particular attention was given to publications addressing preventive, conservative, and endodontic management before, during, and after chemotherapy. Relevant recommendations and clinical practice statements issued by professional organizations, including the Multinational Association of Supportive Care in Cancer (MASCC)/International Society of Oral Oncology (ISOO), the Faculty of Dental Surgery of the Royal College of Surgeons of England, the French Society of Oral Surgery, and the Association Francophone des Soins Oncologiques de Support, were also reviewed. Because this was a narrative rather than a systematic review, the available evidence and recommendations were synthesized qualitatively, and no meta-analysis was performed.

Oral and dental complications associated with chemotherapy

Oral and dental complications may occur during chemotherapy and are significant adverse effects that can directly affect cancer treatment, particularly by increasing the risk of infection in immunosuppressed patients [3].

Oral mucositis

The onset of mucositis a few days after the start of treatment may coincide with neutropenia. It involves painful inflammation of the oral cavity, with erythema followed by irregular ulcers that may spread and become secondarily infected [3-6].

Mucositis appears 4 to 7 days after the start of high-dose treatment and resolves 2-4 weeks after the end of treatment [3-6].

Hyposalivation and xerostomia

This involves direct or indirect damage to the salivary glands, resulting either in a reduction in salivary flow, known as hyposalivation, or, in severe cases, a complete absence of salivary secretion, known as asialia [3,7]. It can lead to numerous complications due to the varied roles of saliva and an imbalance in the oral flora. Xerostomia may contribute to chemotherapy-induced caries [3,4,7,8].

Dysgeusia

Taste disturbances are commonly observed in patients undergoing chemotherapy, causing discomfort. The prevalence of dysgeusia due to chemotherapy has been reported to be 39%. The most common changes include a metallic taste, loss of taste sensitivity, or a bitter taste. These taste alterations may vary according to the chemotherapeutic agents and treatment regimens used. Dysgeusia may develop within a few weeks after the start of treatment and may resolve within hours, days, weeks, or even months after chemotherapy [8,9].

Bleeding

Cytotoxic drugs may suppress bone marrow function and cause thrombocytopenia, thereby increasing the risk of oral bleeding [4]. Petechiae and spontaneous gingival bleeding may indicate a low platelet count. Decisions regarding invasive dental procedures should not be based on a single universal platelet threshold; they should be individualized according to the procedure, the current platelet count and trend, the presence of additional bleeding risks, and advice from the oncology or hematology team [4,10].

Infection

Chemotherapy often causes leukopenia (a reduction in white blood cells), thereby increasing the risk of infection [4,9].

Immunosuppression following cancer treatment (bone marrow failure) makes patients undergoing chemotherapy more susceptible to infections. Consequently, pre-existing or latent oral infections, such as chronic apical periodontitis and periodontal infections, may become symptomatic or be exacerbated during chemotherapy-induced immunosuppression. These infections may act as a portal of entry for bacteria and potentially lead to bacteremia [4,9,11-13].

Chemotherapy-induced caries

Caries are thought to be indirectly linked to xerostomia and chemotherapy-induced changes in the oral flora (resulting from disruption of the salivary glands), as well as to a more cariogenic diet and poor oral hygiene secondary to asthenia. Reduced salivary function may decrease the protective effects of saliva and contribute to an oral environment more favorable to caries development. These combined factors may therefore increase the risk of dental caries during chemotherapy [3,14-16].

Dentin hypersensitivity

This may be linked to a reduction in saliva production. Patients receiving chemotherapy agents based on plant alkaloids (vincristine, vinblastine) may experience deep, constant pain (most commonly affecting the lower molars) in the absence of any proven dental pathology [17].

The pain is usually transient and generally disappears shortly after the dose is reduced and/or chemotherapy is stopped [17].

Medication-related osteonecrosis of the jaw

Medication-related osteonecrosis of the jaw may occur in patients exposed to antiresorptive agents, including bisphosphonates, or certain antiangiogenic agents used as part of cancer care. Bisphosphonates have a strong affinity for bone and may persist in the skeleton for years after treatment has stopped. Clinically, MRONJ may present with exposed necrotic bone or a fistula that probes to bone, with or without pain and infection; non-exposed cases may present with nonspecific symptoms. Radiographic findings may include osteosclerosis, altered trabecular architecture, thickening of the lamina dura, and, in advanced cases, osteolysis or pathological fracture. Differential diagnoses include periodontal or periapical disease, alveolar osteitis, sinusitis, chronic osteomyelitis, and other causes of jaw pain. MRONJ should be distinguished from osteoradionecrosis, which is associated with radiation exposure [18,19].

## Review

General recommendations

Several professional societies have developed recommendations for the oral and dental management of patients undergoing anticancer therapy. These include the French Society of Oral Surgery in 2012 in France [20], the American Cancer Society Head and Neck Cancer Survivorship Care Guideline in 2016 in the United States [21], the British Society for Disability and Oral Health guidelines in the United Kingdom [22], and the Association Francophone des Soins Oncologiques de Support in 2020 [23]. More recently, the Faculty of Dental Surgery of the Royal College of Surgeons of England published comprehensive guidelines entitled The Oral & Dental Management of Patients Before, During and After Cancer Therapy in 2025 [24]. In addition, the Multinational Association of Supportive Care in Cancer and the International Society of Oral Oncology published Clinical Practice Statements in 2024 and 2025 addressing basic oral care and dental evaluation before intensive treatment for hematological malignancies, hematopoietic cell transplantation, and CAR T-cell therapy [25,26].

Despite differences in scope and target populations, these recommendations consistently emphasize that patients should be informed about the general and oral complications associated with anticancer therapy. An early oral and dental assessment should be performed before treatment. Active oral and dental infections should be eliminated or stabilized whenever possible without unnecessarily delaying anticancer therapy. Rigorous oral hygiene should be maintained, and an individualized preventive program should be implemented. Close collaboration between the dentist and the oncology or hematology team should be maintained throughout treatment. Patients should also have access to urgent dental care during treatment and appropriate post-treatment follow-up.

Accordingly, a comprehensive oral health assessment should be systematically performed before the initiation of chemotherapy to identify existing oral disease, assess the patient's oral health status, and establish an appropriate treatment plan.

Patient care before chemotherapy

Recent recommendations emphasize that the dental assessment should be organized as early as possible after the diagnosis of cancer to allow sufficient time for the treatment or stabilization of active oral disease. The objective is to reduce the risk of dental emergencies, pain, and oral infection during anticancer therapy without unnecessarily delaying the start of cancer treatment [24,25,27].

Before chemotherapy, a comprehensive medical history should be obtained, including the initial cancer diagnosis, planned treatments (chemotherapy, radiotherapy, surgery, targeted therapy, or combined modalities), treatment protocols, expected prognosis, hematological and immune status, allergies, and other relevant medical and surgical history. An oral history should also be recorded, including oral hygiene practices, dietary habits, previous dental care, and any history of dental trauma.

A comprehensive oral examination, combined with appropriate radiographic evaluation, should be performed to identify active oral disease and potential sources of infection. An appropriate treatment plan should then be developed in consultation with the oncologist. The urgency, feasibility, and prognosis of conservative and endodontic treatments should be assessed according to the time available before chemotherapy. Teeth requiring urgent treatment because of pain, active pulpal or periapical infection, extensive caries, or a poor restorative prognosis should be identified. An individualized preventive program, including oral hygiene instruction, dietary advice, and fluoride therapy according to the patient's caries risk, should also be implemented [24,25,28-30].

In the presence of poor oral health, the therapeutic approach in conservative dentistry and endodontics should be based on the presence of symptoms or active infection, the restorability and prognosis of the tooth, the expected degree of immunosuppression, and the time available before chemotherapy [24,25,31-33].

Conservative treatment

Active carious lesions should be treated, and defective restorations associated with recurrent caries, loss of coronal seal, or a risk of pulpal complications should be repaired or replaced. When definitive treatment cannot be completed before chemotherapy, the tooth may be temporarily stabilized with a well-sealed restoration.

Vital pulp and endodontic treatment

Teeth presenting with irreversible pulpitis, pulp necrosis, symptomatic apical periodontitis, or an acute apical abscess should be treated as a priority. Root canal treatment should be preferred to extraction when the tooth is restorable, has a favorable prognosis, and treatment can be completed reliably without delaying anticancer therapy [24,25,31,33].

Previously root-filled teeth without pain, swelling, sinus tract, or progressive radiographic disease do not require systematic endodontic retreatment before chemotherapy.

Asymptomatic teeth with a stable periapical radiolucency should be assessed individually. Their management should consider the size and evolution of the lesion, the quality of the root canal filling and coronal seal, the prognosis of retreatment, the expected degree of immunosuppression, and the time available before cancer therapy [24,25,31,33].

The technical principles of nonsurgical root canal treatment are generally similar to those applied to medically healthy patients; however, treatment planning requires particular attention in patients receiving chemotherapy. The timing of dental treatment should take into account the chemotherapy schedule, expected hematological nadir and recovery, and the patient’s general condition. When treatment is required during chemotherapy, the patient’s hematological status, particularly the absolute neutrophil count and platelet count, should be assessed in consultation with the oncology or hematology team [24,25].

Extraction

Extraction should be considered for nonrestorable teeth, teeth with uncontrolled active infection, severe periodontal involvement, vertical root fracture, or a poor endodontic and restorative prognosis [24,25,31,33].

Whenever possible, endodontic treatment should be completed sufficiently early before chemotherapy to confirm the resolution of acute symptoms and to obtain an adequate coronal seal. However, no universal healing interval is applicable to all endodontic procedures, and the timing should be determined according to the urgency of cancer treatment, the clinical condition of the tooth, and the expected degree of immunosuppression [7,12,24,25].

Patient care during chemotherapy

The timing of dental treatment during chemotherapy should be determined according to the treatment protocol, the expected nadir, the recovery of blood cell counts, and the patient’s general condition. There is no single safe time window applicable to every chemotherapy regimen. Whenever possible, elective care should be postponed until hematological recovery. If urgent dental treatment is required, the dentist should consult the oncology or hematology team and obtain a recent complete blood count, particularly the absolute neutrophil count and platelet count [24,25].

During profound neutropenia, severe thrombocytopenia, fever, or clinical instability, elective dental treatment should be delayed. Urgent treatment should be limited to the control of pain and infection and performed in coordination with the medical team, with appropriate antimicrobial or hematological support when indicated [24,25].

When urgent pulpal or periapical disease occurs during chemotherapy, conservative and nonsurgical endodontic approaches should be prioritized whenever they can control pain and infection while avoiding extraction. Treatment may include caries removal and sealing, pulpotomy, pulpectomy, root canal treatment, or drainage through the root canal, depending on the diagnosis and the patient’s medical status. The procedure should be performed with strict aseptic measures, adequate isolation, and minimal trauma. Definitive treatment may be completed after hematological recovery if only emergency treatment is initially possible [24,25,28-30].

Antibiotic prophylaxis is not routinely indicated for all patients receiving chemotherapy or for all conservative and endodontic procedures. Its use should be determined individually according to the absolute neutrophil count, the presence and severity of active infection, the invasiveness of the procedure, the expected duration of immunosuppression, and the recommendations of the oncology or hematology team [25]. In patients with severe neutropenia or profound immunosuppression, urgent invasive treatment should be coordinated with the medical team, which will determine whether antimicrobial coverage, hospital management, or postponement of treatment is required [24,25,29,32,34].

Patient care following chemotherapy

After chemotherapy, the resumption of routine dental treatment depends on the recovery of blood cell counts, immune function, oral mucosal integrity, and the patient’s general condition. Dental care should not automatically be resumed immediately after the final chemotherapy cycle if the patient remains immunosuppressed or thrombocytopenic. When there is uncertainty, the patient’s medical status should be confirmed with the oncology or hematology team [24,25].

Once hematological recovery has occurred, conservative and endodontic treatments may generally be performed according to standard principles. Follow-up should include assessment of caries activity, the integrity of restorations and coronal seals, pulpal and periapical status, periodontal health, salivary function, and persistent oral complications [24,25]. Teeth that received emergency or temporary treatment during chemotherapy should be reassessed and definitively restored or endodontically treated when required.

The frequency of dental follow-up should be individualized according to caries risk, salivary dysfunction, oral hygiene, previous radiotherapy, ongoing immunosuppression, and exposure to antiresorptive or antiangiogenic agents [24,25]. Preventive measures should include fluoride toothpaste, professional fluoride therapy when indicated, dietary counseling, and management of xerostomia. Chlorhexidine should not be prescribed routinely to all patients after chemotherapy but should be reserved for a specific clinical indication [24,26].

## Conclusions

The dentist plays a vital role in the management of patients undergoing chemotherapy before, during, and after treatment. Early dental assessment, elimination of active oral disease, and individualized preventive measures can reduce treatment interruptions, oral infections, and deterioration in quality of life.

Conservative dentistry and nonsurgical endodontic treatment should be preferred whenever the affected tooth has a favorable prognosis. Treatment decisions should be individualized according to the patient's medical status and degree of immunosuppression and should involve close collaboration with the oncology team. Appropriate follow-up after chemotherapy is essential to maintain oral health and ensure long-term treatment success.

## References

[1] Sung H, Ferlay J, Siegel RL, Laversanne M, Soerjomataram I, Jemal A, Bray F (2021). Global cancer statistics 2020: GLOBOCAN estimates of incidence and mortality worldwide for 36 cancers in 185 countries. CA Cancer J Clin.

[2] Harding J (2017). Dental care of cancer patients before, during and after treatment. BDJ Team.

[3] Bonnot J, Pillon F (2013). Chimiothérapie anticancéreuse et prise en charge bucco-dentaire. Actual Pharm.

[4] Kapoor V, Basur S, Pandey A (2015). Chemotherapy and oral complications - the most neglected side of cancer. J Adv Med Dent Sci Res.

[5] J. Aquilina-Arnold, HD et Catherine Grater-Nakamura (2008). HD, la chimiothérapie : considérations pour les hygiènistes dentaire. Can J Dent Hygiene.

[6] Elad S, Cheng KK, Lalla RV (2020). MASCC/ISOO clinical practice guidelines for the management of mucositis secondary to cancer therapy. Cancer.

[7] Hitomi S, Ujihara I, Sago-Ito M, Nodai T, Shikayama T, Inenaga K, Ono K (2019). Hyposalivation due to chemotherapy exacerbates oral ulcerative mucositis and delays its healing. Arch Oral Biol.

[8] Mostafavi S, Wyszyńska M, Skucha-Nowak M (2025). The importance of dental treatment in patients before radiotherapy, chemotherapy, and cardiac surgeries: a narrative review. J Clin Med.

[9] Poulopoulos A, Papadopoulos P, Andreadis D (2017). Chemotherapy: oral side effects and dental interventions - a review of the literature. Stomatol Dis Sci.

[10] Chabolle F, Babin E (2013). Complications et Séquelles des Traitements en Cancérologie ORL: Rapport 2013 de la Société Française d’ORL et de Chirurgie Cervico-Faciale. Issy-les-Moulineaux: Elsevier-Masson.

[11] Houvenaeghel B (2016). Le traitement anticancéreux et ses incidences buccales: prévention et difficultés de traitement. JPIO.

[12] Chaveli Lopez B, Gavalda Esteve C, Sarrion Perez MG (2011). Dental treatment considerations in the chemotherapy patient. J Clin Exp Dent.

[13] Butterworth C, McCaul L, Barclay C (2016). Restorative dentistry and oral rehabilitation: United Kingdom national multidisciplinary guidelines. J Laryngol Otol.

[14] Olczak-Kowalczyk D, Krasuska-Sławińska E, Brożyna A, Turska-Szybka A, Dembowska-Bagińska B (2018). Dental caries in children and adolescents during and after antineoplastic chemotherapy. J Clin Pediatr Dent.

[15] Beacher NG, Sweeney MP (2018). The dental management of a mouth cancer patient. Br Dent J.

[16] Ptasiewicz M, Maksymiuk P, Chałas R (2021). Changes of dentition state in leukemic patients during chemotherapy. Int J Environ Res Public Health.

[17] American Academy of Pediatric Dentistry Dental management of pediatric patients receiving immunosuppressive therapy and/or head and neck radiation. The Reference Manual of Pediatric Dentistry.

[18] Rondot G, Ejeil AL (2007). Evolution de l’ostéonécrose des maxillaires chez les patients sous bisphosphonates: à propos d’un cas. Médecine Buccale Chir Buccale.

[19] Magremanne M, Vervaet C, Dufrasne L, Declercq I, Legrand W, Daelemans P (2006). Bisphosphonates et ostéo(chimio)nécrose maxillo-mandibulaire. Rev Stomatol Chir Maxillofac.

[20] Société Française de Chirurgie Orale (2012). Prise en charge des foyers infectieux bucco-dentaires. Médecine Buccale Chir Buccale.

[21] Cohen EE, LaMonte SJ, Erb NL (2016). American Cancer Society head and neck cancer survivorship care guideline. CA Cancer J Clin.

[22] Kumar N (2019). Updated clinical guidelines on the oral management of oncology patients. Fac Dent J.

[23] Association Francophone des Soins Oncologiques de Support (2020). Traitements médicaux du cancer et soins bucco dentaires. Traitements Médicaux du Cancer et Soins Bucco-Dentaires.

[24] Kumar N, Stockham L, Koshal S (2026). The Oral & Dental Management of Patients Before, During and After Cancer Therapy. The Oral & Dental Management of Patients Before, During and After Cancer Therapy. London: Faculty of Dental.

[25] Zadik Y, Raber-Durlacher JE, Epstein JB (2025). MASCC/ISOO Clinical Practice Statement: dental evaluation and management prior to treatment for hematologic malignancies and CAR T-cell therapy. Support Care Cancer.

[26] Raber-Durlacher JE, Zadik Y, Treister NS, Stempler N, Bruno JS, Epstein JB, Elad S (2024). MASCC/ISOO clinical practice statement: current understanding on controversies in basic oral care in hemato-oncology and hematopoietic cell transplantation. Support Care Cancer.

[27] Yamada SI, Soutome S, Hasegawa T (2020). A multicenter retrospective investigation on the efficacy of perioperative oral management in cancer patients. Medicine (Baltimore).

[28] Brennan MT, Woo SB, Lockhart PB (2008). Dental treatment planning and management in the patient who has cancer. Dent Clin North Am.

[29] Laurent F (2013). Risques Médicaux: Guide de Prise en Charge par le Chirurgien-Dentiste. https://adf.asso.fr/produit/risques-medicaux-guide-de-prise-en-charge-par-le-chirurgien-dentiste/.

[30] Folwaczny M, Hickel R (2001). Aspects of dental care of immunosuppressed patients. I (Article in German). Schweiz Monatsschr Zahnmed.

[31] National Institute of Dental and Craniofacial Research (2009). Prevention and management of oral complications Head and Neck, Radiation Therapy,Chemotherapy, Hematopoietic Stem, Cell Transplantation. In. Dental Provider's Oncology Pocket Guide: Prevention and Management of Oral Complications.

[32] Association Francophone des Soins Oncologiques de Support (2010). Chimiothérapie et Soins Bucco-Dentaires.

[33] Spijkervet FK, Schuurhuis JM, Stokman MA, Witjes MJ, Vissink A (2021). Should oral foci of infection be removed before the onset of radiotherapy or chemotherapy?. Oral Dis.

[34] Agence nationale de sécurité du médicament et des produits de santé (2011). Agence nationale de sécurité du médicament et des produits de santé. Prescription des antibiotiques en pratique bucco-dentaire: recommandations de bonne pratique. Prescription des Antibiotiques en Pratique Bucco-Dentaire: Recommandations de Bonne Pratique.

